# *N*-Acetyl-glucosamine influences the biofilm formation of *Escherichia coli*

**DOI:** 10.1186/s13099-018-0252-y

**Published:** 2018-06-22

**Authors:** Jean-Félix Sicard, Philippe Vogeleer, Guillaume Le Bihan, Yaindrys Rodriguez Olivera, Francis Beaudry, Mario Jacques, Josée Harel

**Affiliations:** 1 Groupe de Recherche sur les Maladies Infectieuses en Production Animale, Centre de Recherche en Infectiologie Porcine et Avicole, Faculté de Médecine Vétérinaire, Université de Montréal, St-Hyacinthe, QC J2S 2M2 Canada; 2 Regroupement de Recherche Pour un Lait de Qualité Optimale (Op+Lait), Faculté de Médecine Vétérinaire, Université de Montréal, Saint-Hyacinthe, QC J2S 2M2 Canada

**Keywords:** *Escherichia coli*, AIEC, Biofilms, Mucus, *N*-Acetyl-glucosamine

## Abstract

**Background:**

The intestinal mucous layer is a physical barrier that limits the contact between bacteria and host epithelial cells. There is growing evidence that microbiota-produced metabolites can also be specifically sensed by gut pathogens as signals to induce or repress virulence genes. Many *E. coli*, including adherent and invasive (AIEC) strains, can form biofilm. This property can promote their intestinal colonization and resistance to immune mechanisms. We sought to evaluate the impact of mucus-derived sugars on biofilm formation of *E. coli*.

**Results:**

We showed that the mucin sugar *N*-acetyl-glucosamine (NAG) can reduce biofilm formation of AIEC strain LF82. We demonstrated that the inactivation of the regulatory protein NagC, by addition of NAG or by mutation of *nagC* gene, reduced the biofilm formation of LF82 in static condition. Interestingly, real-time monitoring of biofilm formation of LF82 using microfluidic system showed that the mutation of *nagC* impairs the early process of biofilm development of LF82. Thus, NAG sensor NagC is involved in the early steps of biofilm formation of AIEC strain LF82 under both static and dynamic conditions. Its implication is partly due to the activation of type 1 fimbriae. NAG can also influence biofilm formation of other intestinal *E. coli* strains.

**Conclusions:**

This study highlights how catabolism can be involved in biofilm formation of *E. coli*. Mucus-derived sugars can influence virulence properties of pathogenic *E. coli* and this study will help us better understand the mechanisms used to prevent colonization of the intestinal mucosa by pathogens.

**Electronic supplementary material:**

The online version of this article (10.1186/s13099-018-0252-y) contains supplementary material, which is available to authorized users.

## Background

*Escherichia coli* is a highly versatile bacterial species commonly found as part of the intestinal microbiota of warm-blooded animals. Most isolates are harmless but some have acquired virulence genes that allow them to cause numerous diseases within the gut (intestinal pathogenic *E. coli*, InPEC) or extra-intestinally (extra-intestinal pathogenic *E. coli*; ExPEC). Commensal *E. coli* colonize the large intestine in vertebrates and appear to reside inside the mucus layer without contacting the underlying epithelium [1]. In contrast, InPEC possess the ability to penetrate the mucus layer and colonize the mucosa causing disease such as diarrhea [2]. Adherent-invasive *E. coli* (AIEC) strains share many genetic and phenotypic features with ExPEC strains but are rather involved in inflammatory bowel disease (IBD), including Crohn’s disease (CD) [3–5].

The mucus layer that covers the intestinal epithelium plays a critical role in gut homeostasis. The intestinal mucus contains mucins which are highly *O*-glycosylated proteins. Mucins play an important role in shaping the intestinal microbiota as an alteration of the glycan availability modifies the microbiota composition [6]. By producing specific glycosidases, several species of the gut microbiota release sugars from *O*-glycans into the intestinal lumen [7, 8]. Released mucus-derived sugars, including *N*-acetylglucosamine (NAG), *N*-acetylneuraminic acid (NANA), galactose, fucose, mannose and *N*-acetylgalactosamine provide direct source of carbohydrates and promote the growth of commensal and pathogenic bacteria including *E. coli* [7–10]. In addition to their role as nutrients, some mucus-derived sugars can act as regulatory signals that influence bacterial colonization and adherence to intestinal cells [11–14]. Enterohemorrhagic *E. coli* (EHEC) uses fucose, NAG and NANA as signaling molecules to modulate its metabolism and regulate the expression of its virulence repertoire [12, 15]. We recently showed that the catabolism of NAG and NANA inhibits EHEC adhesion to epithelial cells through down-regulation of the locus of enterocytes effacement expression under NagC regulatory control [16].

Biofilms could play a key role in bacterial colonization of the healthy gut and in intestinal diseases. Mucin has been reported to be involved in biofilm formation by *E. coli* [17, 18], suggesting its potential role in modulating *E. coli* colonization in the intestinal tract. The ability of biofilm formation in vitro varies extensively among *E. coli* isolates [19] and many *E. coli* strains are believed to form biofilm in the intestinal tract [20]. As such, AIEC are known to be higher biofilm producers than non-AIEC strains [21]. The ability to form biofilm could be part of the etiology of IBD since an increased presence of biofilms formed by the *Bacteroides fragilis* group and the *Enterobacteriaceae* family has been observed in intestinal biopsy specimens of people affected with these diseases [22, 23].

Alteration of the gut mucosal integrity and of microbiota could also change the mucus-derived sugars availability. Our hypothesis is that in healthy conditions, the integrity of the intestinal mucus and the functions of gut microbiota prevent biofilm formation of pathogenic *E. coli*. We evaluated the influence of mucus-derived sugars on biofilm formation of AIEC reference strain LF82. We show that NAG can reduce biofilm formation of LF82 and that the transcriptional regulator of NAG catabolism, NagC appears to be involved in the early steps of its biofilm formation. We also showed that mucus-derived sugars can influence biofilm formation of different *E. coli* strains from other pathotypes.

## Methods

### Bacterial strains and growth conditions

Bacterial strains used in this study are listed in Table 1. Bacteria were routinely cultured on lysogeny broth (LB) agar [1% (wt/vol) tryptone, 0.5% (wt/vol) yeast extract, 1% (wt/vol) NaCl, 1.5% (wt/vol) agar] at 37 °C and single colonies were transferred in liquid LB. When required, the growth medium was supplemented with kanamycin (50 μg/ml) and/or chloramphenicol (25 μg/ml). A set of commensal and pathogenic *E. coli* that comprises AIEC strain LF82, EHEC strain EDL933, enteroagregative *E. coli* (EAEC) strain 17.2, laboratory *E. coli* (K-12) strain MG1655 as well as commensal murine *E. coli* strain NC101 were included in the biofilm studies.

**Table 1:** List of strains and plasmids used in this study

Strains	Characteristics	Origin	Disease	References
LF82	Wild type AIEC O83:H1 AmpR	Human	Crohn’s disease	[4]
LF82Δ*nagC*	LF82 Δ*nagC* AmpR KanR			This study
MG1655	Laboratory *E. coli* K-12	Human	Healthy	[47]
EDL933	Wild type EHEC O157:H7	Human	Diarrhea, HUS	[48]
17.2	Wild type EAEC	Human	Diarrhea	[49]
NC101	Wild type commensal (AIEC-like)	Mouse	Mouse colitis	[50]
χ7213	*thi*-*1 thr*-*1 leuB6 glnV44 fhuA21 lacY1 recA1 RP4*-*2*-*Tc*::Mu *λpir ΔasdA4 Δzhf*-*2*::Tn10			[51]

Plamids	Characteristics	References		
pGEM^®^-T	Cloning vector	Promega Corp., Madison, WI, USA		
pMEG-375	Suicide vector sacRB mobRP4 oriR6K; CmR ApR	Roy Curtiss III, Arizona State University, Tempe, AZ, USA		
pΔ*nagC*	pMEG-375 with Δ*nagC*:: Km^R^	[16]		
pTrc99a	expression vector with IPTG inducible lacI promoter; ApR	[52]		
p*nagC*	pTrc99a with *nagC*	[16]		
p*nagC*-JFS	p*nagC* with Cm^R^	This study		

### Mutagenesis and complementation

The LF82∆*nagC* mutant was constructed by allelic exchange using a suicide vector as described in our previous work [16]. Primers used for mutagenesis are listed in Additional file 1: Table S1. Briefly, the suicide vector pMEG-375 containing the kanamycin resistance cassette from pKD13 flanked with 500 pb sequences upstream and downstream of the *nagC* open reading frame was transformed in a diaminopimelic acid auxotrophic *E. coli* strain χ7213 (λpir and ∆*asdA4*). χ7213 was used as a donor to transfer the plasmid in *E. coli* strain LF82 by conjugation. Single crossover mutants were selected on LB agar without diaminopimelic acid, containing kanamycin. A second selection of double crossovers mutants was made using the *sacB* counter selection on LB agar without NaCl, containing 10% (wt/vol) of sucrose [24]. The mutation of *nagC* was confirmed by PCR and sequencing of an amplicon containing the region of interest. Complementation was performed using a derivative of the expression vector. The ORF of *nagC* was amplified in *E. coli* strain LF82 and inserted downstream of the pTrc promoter in the expression plasmid forming the p*nagC*. A resistance cassette of chloramphenicol was amplified from pACYC184 and inserted in p*nagC*, forming the p*nagC*-JFS.

### Static biofilm assay

The assay of biofilm formation was done in 96-well microtiter plates as previously described [25]. Isolated colonies from LB agar were resuspended in fresh LB (5 ml) and incubated at 37 °C with shaking (180 rpm). Overnight cultures were diluted (1:100) in fresh medium; either LB, LB without salt (LBWS) or M9 medium with 0.4% glucose (wt/vol) and minerals (1.2 mM MgSO_4_, 2 µM FeCl_3_, 8 µM CaCl_2_, and 16 µM MnCl_2_). When required, the growth medium was supplemented with NAG, NANA or fucose (Sigma-Aldrich, St. Louis, MO, USA) at a concentration of 1 mM. A volume of 150 μl of these cultures was deposited in triplicate in the wells. Plates were incubated either at 30 or 37 °C for 24 h, without shaking. After incubation, total growth was measured at OD_630_ with a microplate reader (PowerWave; Bio-Tek Instruments, Winooski, VT, USA). Afterwards, the media were discarded, and each well washed three times with phosphate-buffered saline (PBS) to remove unattached cells. The biofilms were stained with crystal violet 0.1% (wt/vol) for 2 min at room temperature. The crystal violet solution was removed, and the biofilms washed six times with PBS. Finally, an 80% (vol/vol) ethanol and 20% (vol/vol) acetone solution was added to release the stain and the amount of biofilm quantified by measuring at OD_595_. Specific biofilm formation (SBF) index was calculated [21, 26]. Results are the ratio of biofilm measured at OD_595_ over total growth measured at OD_630_. To evaluate the effects of mucus-derived sugars addition on biofilm formation of different strains, one-way ANOVA with Dunnett’s multiple comparison test was performed to calculate *P*-values. To evaluate the impact of the mutation of *nagC* on AIEC strain LF82, two-way ANOVA with Tukey’s multiple comparison test was performed to calculate *P*-values *P <0.05; **P <0.01; ***P <0.001.

### Flow-through biofilm assay (BioFlux device)

The biofilm formation assay in the BioFlux 200 device (Fluxion Biosciences, South San Francisco, CA, USA) was adapted from our previous work [25]. Biofilm formation occurs in the microfluidic channel of the device where fresh media constantly flow through causing a shear force that is similar to physiological condition [25]. *E. coli* strains were isolated on LB agar and resuspended in 5 ml of fresh LB medium. They were incubated overnight at 37 °C with shaking (180 rpm). A 0.5 ml volume of culture was transferred to a microtube and bacteria were collected by centrifugation (13,000*g*, 2 min). LB medium was used to resuspend the pellet to an OD_600_ ≈ 1. Prewarmed LB ± NAG (1 mM) was injected in the microfluidic system to fill the channels. Bacteria were injected from the output reservoir for 30 s at 0.5 dyne/cm^2^. The microfluidic plate was incubated at 30 °C for 2 h to allow the adhesion of bacteria on the surface of the channels. After adhesion, prewarmed LB ± NAG (1 mM) was added to the inlet wells and the microfluidic plate was connected to the BioFlux system. Temperature was adjusted to 30 °C and the flow was initiated at 1.0 dyne/cm^2^. After 4 h, the used medium was removed from the outlet well and prewarmed fresh medium was added in the inlet well. Flow was then reduced at 0.5 dyne/cm^2^ for the next 18 h. Images of the BioFlux biofilms were obtained with an inverted optic microscope equipped with a 40× objective (CKX41; Olympus, Markham, ON, Canada), a digital camera (Retiga EX; QImaging, Surrey, BC, Canada), and the software provided with the BioFlux 200 device. Images were treated using the software imageJ (National Institutes of Health, Bethesda, MD, USA) to quantify the amount of biofilm in the microfluidic channel. The 16-bit grayscale images were adjusted with the threshold function to include the bacterial structure before particle analysis. One-way ANOVA with Dunnett’s multiple comparison test was performed to calculate *P*-values.

### Detection of type 1 fimbriae

The capacity of LF82 and the *nagC* mutant to produce type 1 fimbriae was evaluated by the ability to agglutinate to *Saccharomyces cerevisiae* cells in a mannose-dependant way. As previously described [27], cultures were grown at 30 °C in 20 ml of LB for 24 h without shaking to enhance expression of type 1 fimbriae. NAG was added in the medium at a final concentration of 1 mM. The concentration of an initial suspension of approximately 2 × 10^11^ CFU/ml in PBS was reduced by twofold serial dilutions in microtiter plate (Corning, 2797). An equal volume of a commercial yeast suspension in PBS 3% (wt/vol) (Fleischmann’s Active Dry) was added to each well. After 30 min of incubation at 4 °C, yeast agglutination was observable by precipitation of cells in the wells of the plate. The agglutination titer was recorded as the most diluted bacterial sample giving a positive aggregation reaction. Yeast agglutination was considered dependent of type 1 fimbriae if α-d-mannopyranose 5% (wt/vol) (Sigma-Aldrich) inhibited agglutination. One-way ANOVA with Dunnett’s multiple comparison test was performed to calculate *P*-values.

### Quantitative RT-PCR (qRT-PCR)

Bacteria were precultured as described in previous section. A dilution (1:100) was done in 40 ml of LB ± NAG (1 mM) and bacteria were incubated in polystyrene petri dish overnight at 30 °C. Biofilms were washed with PBS and recovered with a cell scraper. RNA was extracted from biofilm cells using Ambion^®^ RiboPure™-Bacteria Kit (ThermoFisher Scientific, Burlington, ON, Canada), according to the manufacturer’s recommendations. As described in [15], the absence of residual DNA in RNA samples was confirmed by PCR with primers targeting *rpoA*. Complementary DNA (cDNA) was synthetized from 10 μg of RNA, using a reverse transcriptase and random hexanucleotide primers. A standard curve was performed to determine the copy number of targeted transcript in 50 ng of cDNA. Primers used are listed in Additional file 1: Table S1. Results are presented as the ratios between the cDNA copy number of the gene of interest and the cDNA copy number of the housekeeping gene. One-way ANOVA with Dunnett’s multiple comparison test was performed to calculate *P*-values.

## Results

### NAG reduces biofilm formation of AIEC strain LF82

**Fig. 1:**
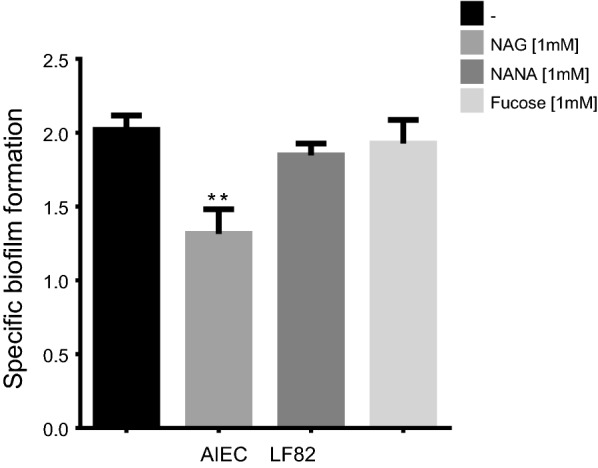
Impact of mucus-derived sugars on specific biofilm formation of AIEC strain LF82. Biofilms of strain LF82 were formed in LB medium under static conditions at 30 °C. NAG, NANA or fucose were supplemented at a concentration of 1 mM. SBF values are the mean and standard error of at least 3 biological experiments. Statistical significance was calculated by one-way ANOVA with Dunnett’s multiple comparison test **P <0.01

We investigated the impact of mucus-derived sugars, including NAG, NANA and fucose, on biofilm formation of the AIEC reference strain LF82. LB culture medium was selected for optimal growth of biofilms in static condition. Upon the addition of 1 mM of NAG, the specific biofilm formation (SBF) index of LF82 was significantly lower (*P* < 0.01) than the SBF index of LF82 grown in LB alone. Addition of NANA and fucose in the medium did not influence the biofilm formation of the strain (Fig. 1). The three mucus-derived sugars did not affect the growth of the strain after 24 h (data not shown). A time course measurement of NAG consumption by LF82 grown in LB supplemented with 1 mM of NAG indicated that NAG was catabolized within 4 h (Additional file 2: Figure S1).

### NAG reduces specific biofilm formation of LF82 by inactivating the transcriptional regulator NagC

**Fig. 2:**
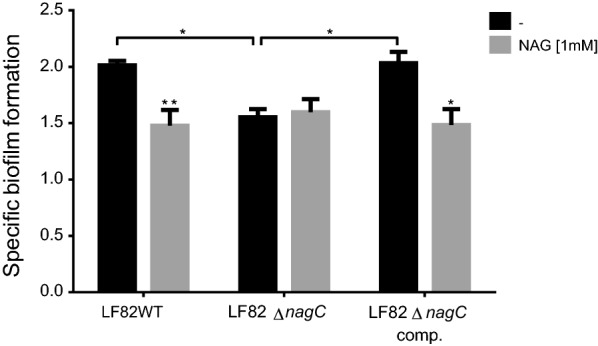
NagC positively influences the biofilm formation of AIEC strain LF82. Biofilms of strain LF82, its isogenic mutant Δ*nagC* and complemented Δ*nagC* were formed in LB medium under static condition at 30 °C. NAG was supplemented at a concentration of 1 mM. SBF values are the mean and standard error of 4 biological replicates. Statistical analysis was calculated by two-way ANOVA with Tukey’s multiple comparison test *P <0.05; **P <0.01

The regulation of AIEC strain LF82 biofilm formation by NAG was further investigated. The activity of the regulator NagC is inactivated by the presence of NAG-6-P, a catabolic derivate of NAG. Therefore, a mutant strain in *nagC* gene was created. The SBF index of LF82Δ*nagC* mutant was significantly lower (*P* < 0.05) than the SBF index of the WT strain. This reduction of biofilm formation was similar to that observed upon the addition of NAG (Fig. 2). The wild-type phenotype was restored in the complemented strain expressing *nagC*. Interestingly, no additional repression was observed upon the addition of NAG in the Δ*nagC* mutant. This indicates that LF82 biofilm repression by NAG is NagC dependent.

### NagC is involved in the first steps of biofilm formation of LF82 in presence of a shear force

**Fig. 3:**
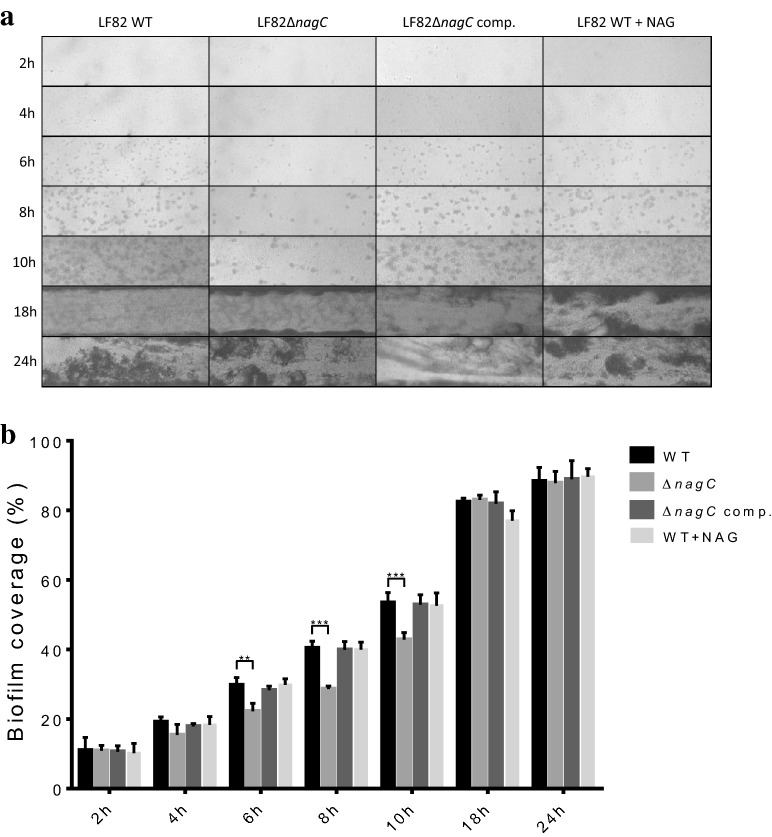
The biofilm formation is delayed in *nagC* mutant of LF82 in a microfluidic system. **a** Biofilms of strain LF82, its isogenic mutant Δ*nagC* and complemented Δ*nagC* were formed in LB medium at 30 °C in the BioFlux 200 microfluidic system. NAG was supplemented at a concentration of 1 mM. A field of view representative of 3 independent replicates is shown for each test. Images were captured by phase contrast microcopy using a digital camera. **b** Quantification of biofilm formation was made using the “Analyze Particles” function of ImageJ and results show the percentage of the area covered by biofilm structures in images pictures. Statistical significance was calculated by one-way ANOVA with Dunnett’s multiple comparison test **P 0.01; ***P 0.001

To further investigate the role of NagC on biofilm formation of LF82, the dynamics of biofilm formation were monitored and visualized in real time using the BioFlux device (Fig. 3a). Real time imaging with LF82 clearly revealed the apparition of microcolonies 6 h after the initiation of the flow. These microcolonies grew overtime and the production of polymeric matrix became more apparent at 10 h. After 18 h, the channel was almost entirely covered with biofilm. At 24 h, mature biofilms were formed. The kinetics of biofilm formation of LF82Δ*nagC* was different from that of the WT LF82. In early time points, a reduced number of microcolonies was observed in the mutant strain. Quantification results revealed a significant (*P* < 0.01) decrease in biofilm coverage of the mutant strain when compared to the WT strain at 6, 8 and 10 h after the activation of the flow. At 16 and 18 h, the biofilm structure of LF82Δ*nagC* mutant was similar to that of the WT. Again, wild-type phenotype was restored in the complemented strain (Fig. 3a, b). This suggests that the *nagC* mutation impairs the first steps of biofilm formation of LF82 and involves surface adhesion and/or cell to cell interactions. In contrast to what we observed in static condition, supplementation of NAG did not affect biofilm formation in the microfluidic system.

### The mutation of *nagC* impaired the production of type 1 fimbriae of LF82

**Fig. 4:**
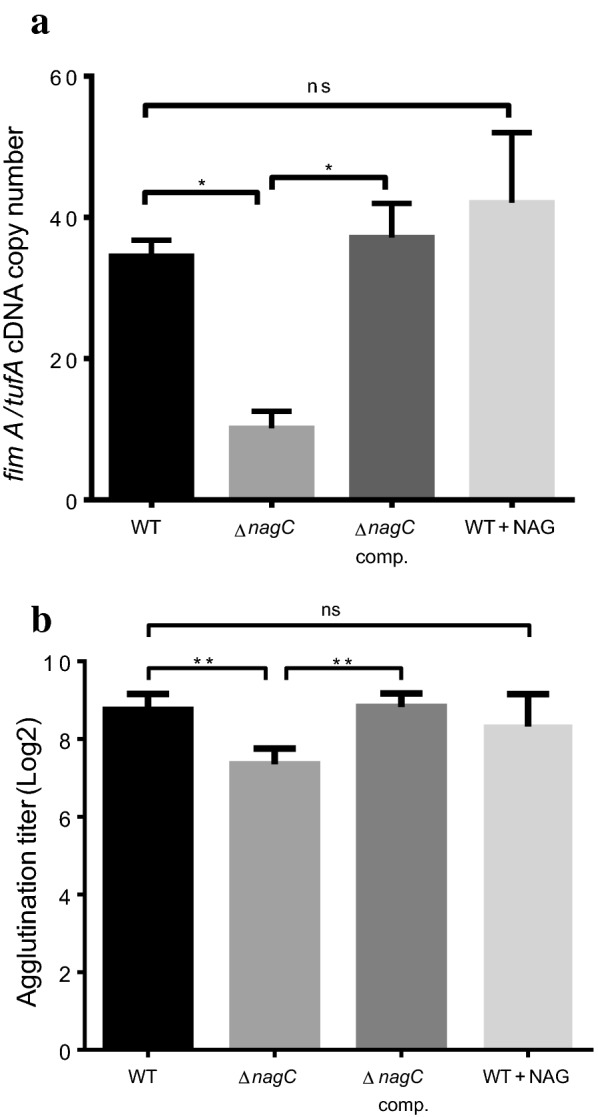
NagC influences the expression and the production of type 1 fimbriae of LF82. **a** The expression of type 1 fimbriae subunit *fimA* was measured by qRT-PCR in LF82 WT strain, its Δ*nagC* derivative and the complemented mutant grown as biofilm in LB media. NAG was also supplemented at a concentration of 1 mM in media used for WT strain. Results are shown as the ratio of copy number of *fimA* transcript/copy numbers of *tufA* transcript. **b** Production of type 1 fimbriae was also evaluated for each strain by mannose specific agglutination to the yeast *S. cerevisiae*. LB medium was used in the experiments and NAG was supplemented at a concentration of 1 mM when needed. Results are the mean values and standard error of 3 biological experiments. Statistical analyzes were made using one-way ANOVA with Dunnett’s multiple comparison test *P 0.05; **P 0.01

To determine the role of type 1 pili in the NagC-dependent reduction of biofilm formation of LF82, the expression and the production of type 1 fimbriae were evaluated in the mutant strain. The expression of *fimA*, encoding the major structural subunit of type 1 fimbriae, was measured in grown biofilms (Fig. 4a). Results showed a significant (*P* < 0.01) reduction of 3.5-fold of *fimA* transcription in the LF82Δ*nagC* strain when compared to WT strain (Fig. 4a). The expression of *fimA* was restored in the complemented strain. However, the effect of supplementation of NAG in medium on the expression of *fimA* was not statistically significant but slightly increased (Fig. 4a). The production of type 1 fimbriae was measured by mannose-sensitive agglutination to *S. cerevisiae* and was significantly lower (*P* < 0.01) by 2 Log_2_ in LF82Δ*nagC* mutant in comparison to WT strain (Fig. 4b). Type 1 pili production was restored in the complemented strain. Addition of NAG during growth did not affect the production of type 1 fimbriae. Interestingly, NAG is rapidly consumed within 4 h of bacterial growth in LB medium supplemented with NAG, as measured by mass spectrometry (Additional file 2). Thus, the repressive effect of NAG on type 1 fimbriae production might be transient. Addition of mannose was previously reported to reduce the biofilm of LF82 by interfering with type 1 fimbriae adhesion [40]. In presence of mannose, biofilm formation of LF82 was decreased in either LB or NAG supplemented medium indicating that type 1 fimbriae is involved in biofilm in these conditions (Additional file 3: Figure S2).Taken together these results show that expression and production of type 1 fimbriae are regulated by NagC in LF82. Type 1 fimbriae or other structures participating to LF82 biofilm might be influenced by NAG supplementation.

### The impact of mucus-derived sugars on biofilm formation varies among *E. coli* strains

**Fig. 5:**
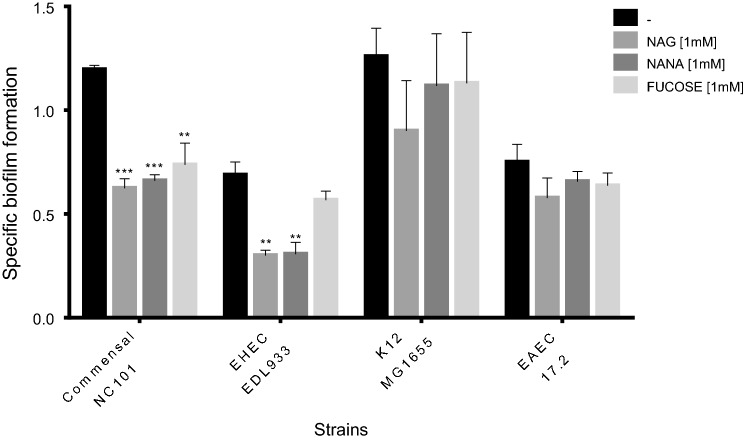
Mucus-derived sugars can influence the specific biofilm formation of different strains of *E. coli*. Strain specific optimized conditions for biofilm formation were identified. LB was used for EAEC strain 17.2 and K-12 strain MG1655. LBWS was used for AIEC strain NC101 and M9 with glucose was used for EHEC strain EDL933. Biofilms were formed under static conditions at 30 °C except for strain EDL933 that was grown at 37 °C. NAG, NANA and fucose were supplemented at a concentration of 1 mM. SBF values are the mean and standard error of at least 3 biological experiments. Statistical analysis was made using one-way ANOVA with Dunnett’s multiple comparison test *P 0.05; **P 0.01; ***P 0.001

Biofilm formation of a set of distinct *E. coli* strains was tested in different culture media to determine optimal conditions. LB medium was used for strains 17.2 and MG1655, whereas LBWS and M9 with glucose were used as the optimized culture media for biofilm formation of strains NC101 and EDL933, respectively. The effect of addition of NAG, NANA or fucose on biofilm formation was evaluated in static condition. The influence of mucus-derived sugars was variable, strain-dependent and did not affect the growth of bacteria as measured with optical density (Additional file 4: Figure S3). Our data showed that the mean OD value of biofilm formed by murine strain NC101 was significantly decreased in the presence of the all three sugars (*P* < 0.01). Supplementation of the medium with NAG (*P* < 0.01) or NANA (*P* < 0.05), significantly reduced biofilm formation of EHEC strain EDL933. A slight reduction was also observed upon the addition of NAG in EAEC strain 17.2 and K-12 strain MG1655 (Fig. 5).

## Discussion

There is growing evidence that microbiota-produced metabolites can also be specifically sensed by pathogens as signals to induce or repress virulence genes [28]. We show that the mucin sugars NAG, NANA and fucose can reduce the biofilm formation of AIEC strain LF82 and other pathogenic *E. coli*. A recent study showed that fucose modulated biofilm formation of *Campylobacter jejuni* [29]. We observed that the influence of mucus-derived sugars on biofilm formation was strain-dependent, reflecting the high genetic diversity and the variability of metabolic patterns between *E. coli* strains [30, 31]. It is known that *E. coli* preference for mucus-derived sugars varies from one strain to another [10, 32, 33]. Interestingly, the repressor effect of NAG was common among the tested *E. coli* strains as NAG supplementation reduced biofilm formation of AIEC strain LF82, murine strain NC101, EHEC strain EDL933 and to a lower extent EAEC strain 17.2 and K-12 strain MG1655.

We demonstrated that the effect of NAG on biofilm formation of AIEC strain LF82 is NagC-dependent. NagC is a repressor of *nag* operon involved in catabolism of NAG in *E. coli* [34, 35]. The uptake of this sugar leads to the production of intracellular NAG-6-P that will inactivate the regulator NagC [36]. Our study shows that the inactivation of NagC, whether it is caused by the catabolism of NAG or by a mutation of *nagC*, is responsible for the decreased biofilm formation. Thus, NagC is a positive regulator of biofilm formation in LF82. Interestingly, NagC is also involved in the expression of the locus of enterocytes effacement virulence genes of EHEC as well as type 1 fimbriae of *E. coli* K-12 [13, 16, 36].

The real-time monitoring of LF82 biofilm formation using microfluidic system showed that early steps are impaired by the mutation of *nagC*. Biofilm formation initially required the attachment of the bacteria to a surface and the cell-to-cell adhesion that leads to the formation of microcolonies. Thus, during these steps, NagC might influence the expression of structures involved in early adhesion of bacteria. In contrast to the situation in static conditions, the biofilm in dynamic conditions was insensitive to the presence of NAG. It is possible that constant renewal of media in dynamic conditions influences NAG catabolism and thus NagC activity.

Type 1 fimbriae are key factors that facilitate adhesion to a surface and cell-to-cell aggregation during establishment of biofilm on abiotic surfaces [37, 38]. They also participate in biofilm formation and in the adhesion-and-invasion process in AIEC strains such as LF82 [21, 39, 40]. In the present study, we show that NagC activates the gene expression and the production of type 1 fimbriae of AIEC strain LF82. This is similar to the work showing NagC regulation on type 1 fimbriae in *E. coli* K-12 MG1655 [13]. Based on NagC consensus DNA binding site generated from known NagC binding sequences [13, 16, 41, 42], two different binding sites were found upstream of the promoter of *fimB* recombinase in LF82. FimB is involved in the OFF-to-ON switching of type 1 fimbriation and sequences found in LF82 were identical and in the same distance to those of *fimB* promoter in K-12 strain MG1655 [13] (Additional file 5: Figure S4). This indicates that NagC control on type 1 fimbriae could influence at least in part the biofilm formation of LF82. In contrast, NAG supplementation did not influence type 1 fimbriae production of LF82 strain. It is possible that NAG-dependant repression on type 1 fimbriae was transient because NAG is rapidly consumed by the strain). NAG might also influence other factors contributing to biofilm formation of LF82 as NAG was also shown to influence the production of curli in *E. coli* K-12 [11].

Dysbiosis that occurs during IBD can favor AIEC growth and probably biofilm formation [43]. As glycosylation of the mucin is defective in CD [44] and microbiota activity is modified, it is possible that the availability of mucin sugars will influence not only the metabolic activity but also the virulence behavior including the pathogens’ ability of biofilm formation. Thus, factors that regulate biofilm formation could signal to repress expression of the type 1 fimbriae and other factors contributing to biofilm of LF82. By affecting the concentration of free NAG available in the digestive tract, gut bacterial species expressing *N*-acetylglucosaminidase [6] might therefore influence *E. coli* biofilm formation through a modulation of NagC activity. Interestingly, administration of glucosamine can reduce production of pro-inflammatory cytokines and therefore intestinal inflammation in murine model of IBD could influence the activity of the flora including AIEC and reduce their biofilm formation and colonization ability [45, 46].

## Conclusion

In conclusion, the presence of mucin-derived sugars can influence biofilm formation of different *E. coli* strains. This study highlights that the diminution of biofilm formation of AIEC strain LF82 in the presence of NAG is NagC-dependent. Indeed, the NAG sensor NagC is involved in the early steps of biofilm formation of strain LF82 and its implication could be partly due to the control of type 1 fimbriae.

## Additional files

**Additional file 1: Table S1.** List of primers used in this study.

**Additional file 2: Figure S1.** Monitoring of NAG consumption by LF82 WT, LF82Δ*nagC* and the complemented strain when grown under static condition. Concentration of NAG was measured by mass spectrometry.

**Additional file 3: Figure S2.** Specific biofilm formation of LF82 was evaluated in the presence or absence of mannose in either in LB or LB supplemented with NAG.

**Additional file 4: Figure S3.** The effect of the addition of mucus-derived sugars on growth of strains.

**Additional file 5: Figure S4.** NagC consensus DNA binding sites and nucleotide BLAST of LF82 and MG1655.

## Abbreviations

AIEC: adherent and invasive *E. coli*
NAG: *N*-acetyl-glucosamine
InPEC: intestinal pathogenic *E. coli*
ExPEC: extra-intestinal pathogenic *E. coli*
IBD: inflammatory bowel disease
CD: Crohn’s disease
NANA: *N*-acetylneuraminic acid
EHEC: enterohemorrhagic *E. coli*
LB: lysogeny broth
EAEC: enteroagregative *E. coli*
LBWS: LB without salt
PBS: phosphate-buffered saline
OD: optical density
SBF: specific biofilm formation
cDNA: complementary DNA
