# Supplementary material for: N-Acetyl-glucosamine influences the biofilm formation of Escherichia coli
Source: Gut Pathog. 2018 Jun 22;10:26. doi: 10.1186/s13099-018-0252-y (PMC6013987; doi:10.1186/s13099-018-0252-y)
Supplement: Supplementary file 1 — Additional file 1: Table S1. List of primers used in this study. [file 13099_2018_252_MOESM1_ESM.docx]

**Table S1. List of primers used in this study**

| Names | Sequence 5’ 🡪 3’ | Use |
| --- | --- | --- |
| M-*tufA*-For | GAAGAAGTTGAAATCGTTGG | Standard curve |
| M-*tufA*-Rev | GAAGAAGTTGAAATCGTTGG | Standard curve |
| q-*tufA*-For | GTGAAGAAATCGAACGTG | qRT-PCR |
| q-*tufA*-Rev | TTCAATGGTACCAGTCAC | qRT-PCR |
| M-*fimA*-For | CTCTGTCCCTGAGTTCTACA | Standard curve |
| M-*fimA*-Rev | ATTAGCAGCACCTGGGGTTG | Standard curve |
| q-*fimA*-For | GGCTCTGTTGATCAAACCGT | qRT-PCR |
| q-*fimA*-Rev | CCGCAGCTGAACTCTGTAGA | qRT-PCR |
| *nagC*-aval-For | ACTTTCTCTTATTGAGTTACGACCTCGTTA | Construction of *nagC* mutant |
| *nagC*-amont-Rev | TGTGCTTTTATAGTGGCGCTTATTGTTGTC | Construction of *nagC* mutant |
| AscI-*nagC*-For | AGGCGCGCCTACCGCGCCCGTGGGTGTCCG | Construction of *nagC* mutant |
| SacI-*nagC*-Rev | CGAGCTCGGCGATGCCGTATATTACCGG | Construction of *nagC* mutant |
| H2P2-*nagC*-For | AGCGCCACTATAAAAGCACAtgtaggctggagctgcttcg | Construction of *nagC* mutant |
| H1P1-*nagC*-Rev | GTAACTCAATAAGAGAAAGTattccggggatccgtcgacc | Construction of *nagC* mutant |
| *nagC*-comp-For-SacI | CGAGCTCGTAACGAGGTCGTAACTCAAT | *nagC* mutant complementation |
| *nagC*-comp-Rev-BamHI | CGGGATCCCGGCGCCACTATAAAAGCACAT | *nagC* mutant complementation |
