# Supplementary material for: N-Acetyl-glucosamine influences the biofilm formation of Escherichia coli
Source: Gut Pathog. 2018 Jun 22;10:26. doi: 10.1186/s13099-018-0252-y (PMC6013987; doi:10.1186/s13099-018-0252-y)
Supplement: Supplementary file 2 — Additional file 2: Figure S1. Monitoring of NAG consumption by LF82 WT, LF82ΔnagC and the complemented strain when grown under static condition. Concentration of NAG was measured by mass spectrometry. [file 13099_2018_252_MOESM2_ESM.docx]

**Mass Spectrometry**

The concentrations of NAG were determined using a High-Performance Liquid Chromatography-High-Resolution, Accurate-Mass Mass Spectrometry (HPLC-HRAM MS) assay. 50 μL of samples were mixed with 500 μL of internal standard solution (100 µM of stable isotope labeled products of NAG). The samples were then vortexed vigorously and centrifuged at 12,000 *g* for 10 min and 200 μL of the supernatant was transferred into an injection vial. Ten µL of samples were injected with a Thermo Scientific UltiMate 3000 Rapid Separation UHPLC system (San Jose, CA, USA) onto a Thermo Biobasic C18 5 x 1 mm column (5 µm) with a flow rate of 100 µL/min. The mobile phase consisted of a mixture of methanol and 20 mM ammonium acetate aqueous solution (pH ≈ 7.0) at ratio of 50:50. The Thermo Scientific Q-Exactive Orbitrap Mass Spectrometer (San Jose, CA, USA) mass spectrometer (Concord, ON, Canada) interfaced with the HPLC system used a pneumatic assisted electrospray ion source operating in positive ion modes. Nitrogen was used for sheath and auxiliary gases and they were set at 10 and 5 arbitrary units. The heated ESI probe was set to 4000 V and the ion transfer tube temperature was set to 300°C. Data was acquired at a resolving power of 140,000 (FWHM using automatic gain control target of 3.0x10^6^ and maximum ion injection time of 200 msec in targeted SIM mode. Ion were extracted using accurate mass of the [M+H]^+^ ion with a ± 5 ppm window for NAG and ^13^C_2_-NAG. The peak area ratios of the light- and heavy-isotopic pairs were used for quantification.

**Figure S1. Monitoring of NAG consumption by LF82 WT, LF82ΔnagC and the complemented strain when grown under static condition.** Strains were incubated at 30°C in LB media containing 1 mM of NAG. Samples of media were taken and filtered at different time points. Concentration of NAG was measured by HLPC-SRM MS. Growth was evaluated by optical density at 600nm.
