# Supplementary material for: N-Acetyl-glucosamine influences the biofilm formation of Escherichia coli
Source: Gut Pathog. 2018 Jun 22;10:26. doi: 10.1186/s13099-018-0252-y (PMC6013987; doi:10.1186/s13099-018-0252-y)
Supplement: Supplementary file 3 — Additional file 3: Figure S2. Specific biofilm formation of LF82 was evaluated in the presence or absence of mannose in either in LB or LB supplemented with NAG. [file 13099_2018_252_MOESM3_ESM.docx]

**The effect of competition with mannose**

Specific biofilm formation of LF82 was evaluated in the presence or absence of mannose in either in LB or LB supplemented with NAG. Statistical analysis was made using one-way ANOVA with Dunnett’s multiple comparison test *, *P* 0.05; **, *P* 0.01.


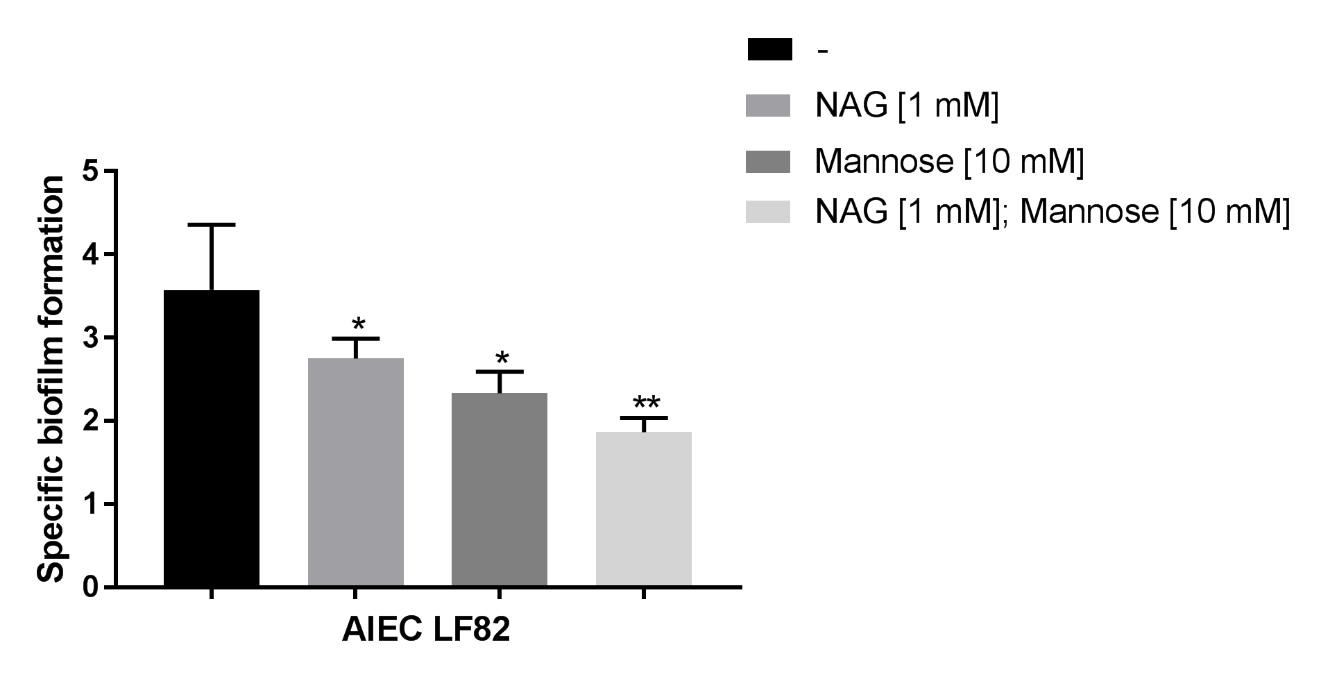


**Figure S2. The effect of competition with mannose is similar to the addition of NAG on the reduction of the biofilm formation of LF82.**

Type 1 pili attach to cells and surfaces in a mannose-sensitive manner. Competition with mannose reduced specific biofilm formation by LF82 in LB to a level similar to that of NAG supplemented medium.
