# Supplementary material for: N-Acetyl-glucosamine influences the biofilm formation of Escherichia coli
Source: Gut Pathog. 2018 Jun 22;10:26. doi: 10.1186/s13099-018-0252-y (PMC6013987; doi:10.1186/s13099-018-0252-y)
Supplement: Supplementary file 4 — Additional file 4: Figure S3. The effect of the addition of mucus-derived sugars on growth of strains. [file 13099_2018_252_MOESM4_ESM.docx]

**Evaluation of growth**

Growth of strains was evaluated in the same media used for biofilm formation. Overnight cultures were diluted (1/100) in 5 ml of fresh media containing 1mM of different mucus-derived sugars. A volume of 200 μl was transferred in polystyrene 96 well plate and incubated under static condition at 30°C with the exception of EDL933 at 37°C. OD values are the mean and standard error of at least 3 biological experiments. Statistical analysis was made using one-way ANOVA with Dunnett’s multiple comparison test.

**Figure S3. The effect of the addition of mucus-derived sugars on growth of strains.**
