# Supplementary material for: N-Acetyl-glucosamine influences the biofilm formation of Escherichia coli
Source: Gut Pathog. 2018 Jun 22;10:26. doi: 10.1186/s13099-018-0252-y (PMC6013987; doi:10.1186/s13099-018-0252-y)
Supplement: Supplementary file 5 — Additional file 5: Figure S4. NagC consensus DNA binding sites and nucleotide BLAST of LF82 and MG1655. [file 13099_2018_252_MOESM5_ESM.docx]

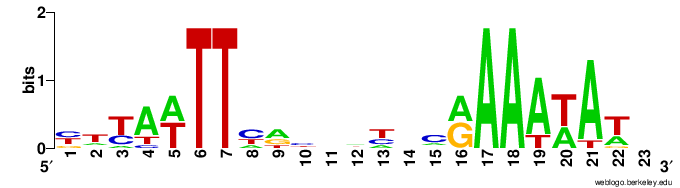


**Figure S4. NagC consensus DNA binding site generated by Weblogo from known NagC binding sequences [13, 16, 41, 42]**.

Two different binding sites were found upstream of the promoter of *fimB* recombinase in LF82. These sequences were identical to those of *fimB* promoter described in K-12 strain MG1655 [13]. BLAST was generated from NCBI.

**LF82** AAGTGATGTTTGCCATAGATTGCGAGATGCCCTGTCTCATTGACTATCGCGGTGCACCTT

|||||||||||||||||||||||||||||||||||||||||||||||| ||||||||||

**MG1655** AAGTGATGTTTGCCATAGATTGCGAGATGCCCTGTCTCATTGACTATCTTGGTGCACCTT

**LF82** TATACCTGTTATACCAGATCAAAAATCACGCAATCCATACAACAAAACGAGATTTGCAAT

|||||||||||||||||||||||||||||||||||||||||||||||| |||||||||||

**MG1655** TATACCTGTTATACCAGATCAAAAATCACGCAATCCATACAACAAAACCAGATTTGCAAT

**LF82** TCGTGTCACAAAATATGTCGATCTTTTTCTAAGAGGAAGATGCCATGTGAAGCCAGACGA

||||||||||||||||||||||||||||||||||||||||||||||||||||||||||||

**MG1655** TCGTGTCACAAAATATGTCGATCTTTTTCTAAGAGGAAGATGCCATGTGAAGCCAGACGA

**LF82** ACACTTGCGGTGGTCTTCAAAAACTAAAGGCATTCTTTTAACTATTTGTTTTATAAATGA

||||||||||||||||||||||||||||| || |||||||||||||||||||||| |

**MG1655** ACACTTGCGGTGGTCTTCAAAAACTAAAGATCTTAGTTTAACTATTTGTTTTATAAATAA

**LF82** TTTATTTGGGGTCTAAACAAGGGAAGCTTTGCAAGCTAGCTCAGTGAGCGTGGTGAAAAT

|||||| | ||||||||||||| |||||||||||||| |||||||||| ||||||||||

**MG1655** TTTATTAAGAGTCTAAACAAGGGGAGCTTTGCAAGCTAACTCAGTGAGCTTGGTGAAAAT

**LF82** AAGTGTTTACCCGCCATCAGGCTGATCATAATTCTCATCATGAAATATGTTTCCTGGTTT

|||||||||||||||||||||||| ||||||||||||||||||||||||||||||||||

**MG1655** CAGTGTTTACCCGCCATCAGGCTGAGCATAATTCTCATCATGAAATATGTTTCCTGGTTT

**LF82** TTGGCTTGTAAGTGGTCACCCCTGAAGTCGATCTGGAAAGGCTTGTAGATGTTGGTGTTT

|||||||||| ||||||| |||||||||||||||| |||||||| |||||||||||||

**MG1655** GTGGCTTGTAACTGGTCACTTCTGAAGTCGATCTGGAGAGGCTTGTTGATGTTGGTGTTT
